# Silencing Chitinase Genes Increases Susceptibility of* Tetranychus cinnabarinus* (Boisduval) to Scopoletin

**DOI:** 10.1155/2017/9579736

**Published:** 2017-12-31

**Authors:** Hong Zhou, Yong-qiang Zhang, Ting Lai, Dan Wang, Jin-lin Liu, Fu-you Guo, Wei Ding

**Affiliations:** Laboratory of Natural Products Pesticides, College of Plant Protection, Southwest University, Chongqing 400715, China

## Abstract

The carmine spider mite* Tetranychus cinnabarinus *is a major pest of crop and vegetable plants worldwide. Previous studies have shown that scopoletin is a promising acaricidal compound against* Tetranychus cinnabarinus. *However, the acaricidal mechanism of scopoletin remains unclear. In the present study, 12 full-length cDNAs of chitinase (CHIT) genes from* Tetranychus cinnabarinus *(designated* TcCHITs*) were cloned and characterized. Although* TcCHITs *were expressed throughout all life stages, their expression levels were significantly upregulated during the larval and nymphal stages.* TcCHITs *were downregulated 24 h after treatment with scopoletin and upregulated 24 h after treatment with diflubenzuron (DFB, a chitin synthesis inhibitor). Feeding double-stranded RNA effectively silenced* TcCHIT *transcription in* Tetranychus cinnabarinus*, thus increasing its susceptibility to scopoletin but reducing that to DFB. Meanwhile,* TcCHIT* silencing in larvae and adult resulted in an extremely low molting rate (7.3%) and high mortality rate (53.3%), respectively, compared with those in the control group. CHIT genes are closely related to arthropod survival, molting, and development in* Tetranychus cinnabarinus*, suggesting that acaricidal mechanisms of scopoletin and DFB may occur by inhibition and activation of CHIT gene expression, respectively.* TcCHIT* constitutes a possible target of scopoletin and DFB in* Tetranychus cinnabarinus*.

## 1. Introduction

Phytophagous mites of the genus* Tetranychus* and* Panonychus* are major pests on plants worldwide [1, 2]. The carmine spider mite* Tetranychus cinnabarinus* is of particular importance, because this extreme generalist species has been documented on more than 100 plant species, including food and economic crops, ornamental plants, and weeds [3–5]. Cinnabar spider mite is parthenogenic and exhibits strong fecundity and adaptability. This mite is also one of the pests that are most difficult to control because it easily develops resistance to pesticides [6].

Control of* Tetranychus cinnabarinus *in open-field crops primarily relies on synthetic chemical acaricides [2, 7–9]. Chemical acaricides have been extensively used to control mite pests because of their quick and efficient acaricidal effect [10]. However, spider mites rapidly develop resistance to almost all acaricidal agents, presenting a major factor that threatens efficient control of spider mites in agriculture [11, 12]. Furthermore, application of chemical acaricides has led to environmental and human health concerns [13]. Thus, controlling mite pests by traditional chemical acaricides has become challenging. Effective methods for controlling mite pests and environment-friendly acaricides should be developed. Phytogenous acaricides, which present low mammalian toxicity and can be rapidly degraded, are suitable for integrated mite management. Studies have also shown that these naturally occurring products may delay development of pesticide resistance in pests [14, 15].

Scopoletin, a coumarin compound, is an important secondary plant metabolite and phytogenous acaricidal compound with excellent contact-killing, systemic, repellent, and oviposition inhibition activities against* Tetranychus cinnabarinus *[16]. Studies have confirmed that scopoletin manifests growth-regulating, insecticidal, and antibacterial activities [17, 18]. The biological functions influenced by scopoletin are attributed to its various molecular targets, including transcription factors, growth factors, and their receptors, cytokines, enzymes, and genes that regulate cell proliferation and apoptosis [19]. Thus, understanding the mode of action of acaricides is crucial to identifying their molecular targets [20]. Although the acaricidal activity of scopoletin and its possible biochemical mechanism have been investigated, its molecular mechanism or molecular target(s) against* Tetranychus cinnabarinus* remains unknown.

Chitin is a polymer of *β*(1,4)-linked N-acetylglucosamine, which is the second most abundant natural polymer after cellulose. Chitin is extensively distributed as a structural component in arthropods, parasites, and microbes [21, 22]. Chitin is also the principal structural component of arthropod exoskeletons and the peritrophic membrane (PM) that lines the epithelium and envelopes gut contents [23]. New chitin is deposited and synthesized during insect growth and development. However, a part of the old cuticle is degraded. When insects or mites are treated with chitin synthesis inhibitors, such as diflubenzuron (DFB) and allosamidin, symptoms of death include development retardation, dysecdysis, and shrinkage [24, 25]. Findings showed that mites treated with scopoletin also exhibit similar death symptoms (Figure 6(c)) [26].

In arthropods, the crucial step of chitin biodegradation pathway is associated with chitinases (CHITs). Insect CHITs, which belong to family 18 of glycosyl hydrolases, mediate digestion of chitin to chitooligosaccharides by hydrolyzing chitin via endo-type cleavage. CHITs are crucial in growth and development of insects and mites and act by hydrolyzing chitin of insect integument and midgut. A total of 16, 22, 20, and 12 CHIT and CHIT-like protein genes have been identified in* Drosophila melanogaster*,* Anopheles gambiae*,* Tribolium castaneum*, and* Tetranychus urticae*, respectively [27]. These genes have been classified into five or more groups on the basis of amino acid sequence similarity and phylogenetic analyses. CHIT and related proteins influence molting, digestion, cell proliferation, and tissue remodeling of mites and insects. Research showed that chitin is a critical component of insect cuticle and PM and that each period of growth and development of insects and mites requires a certain amount of chitin [23]. CHIT is a safe target of novel biological pesticides because chitin is absent in animals and plants [28]. CHIT inhibitors, such as allosamidin, argifin, and argadin, exert insecticidal effects by inhibiting CHIT activity, which interferes with normal growth and development of insects and mites [25, 29, 30]. Scopoletin has been reported for its insecticidal and growth inhibitory effects against* Plutella xylostella*,* Spilarctia obliqua*, and* Diabrotica beetles* [5, 17, 31]. In recent years, experimental evidence has shown that scopoletin inhibits the development of* Tetranychus cinnabarinus*, indicating that this compound may affect degradation of chitin by regulating expressions of CHIT genes [32].

Our laboratory investigated the transcriptomics of* Tetranychus cinnabarinus *after treatment with scopoletin or a control solvent (Table S1 and S2, resp.). Interestingly, we observed that the CHIT gene family, which may be involved in the acaricidal mechanism of scopoletin against* Tetranychus cinnabarinus*, was differentially expressed. Thus, this study aimed to assess the role of differential expression of CHIT mRNA transcripts in acaricidal activity of scopoletin against* Tetranychus cinnabarinus* using RNA interference (RNAi). RNAi is a common mechanism of gene silencing in eukaryotic organisms. In recent years, this technique has shown considerable potential in controlling insect pests by silencing vital genes. Functional roles of CHIT genes in* Tribolium castaneum *and* Panonychus citri* were also evaluated using RNAi, and several CHIT genes were found to be essential for insect survival, molting, and development [33, 34], thereby revealing functional specialization among CHIT genes during molting. Thus, developing synthetic inhibitors that target CHITs is important for insect pest management.

In the present study, 12 full-length cDNAs of CHIT genes from* Tetranychus cinnabarinus* were cloned and characterized. Patterns of gene expressions in four developmental stages upon acaricidal treatment were analyzed. We adopted double-stranded RNA (dsRNA) feeding as gene knockdown strategy to investigate the role of CHITs in the acaricidal action of scopoletin and DFB, a chitin synthesis inhibitor, against* Tetranychus cinnabarinus*. This study demonstrated that suppressing* TcCHIT *transcription increases susceptibility of* T. cinnabarinus* to scopoletin but reduces that to DFB. This study also clarified the role of CHITs in the acaricidal mechanism of scopoletin against* Tetranychus cinnabarinus.*

## 2. Materials and Methods

### 2.1. Mite Rearing

The* Tetranychus cinnabarinus *colony used in this study was collected from cowpeas in Beibei, Chongqing, China, and has been maintained for more than 16 years without exposure to any pesticides [35]. The mites were reared on potted cowpea seedlings* (Vigna unguiculata)* in the insectary at 25 ± 1°C, 50% ± 5% RH, and 14 : 10 h (L : D) photoperiod.

### 2.2. Bioassays and RNA-seq Data

Food and Agriculture Organization-recommended slip-dip method was used to measure scopoletin (purity, 95%; Southwest University, Beibei, Chongqing, China) and DFB (purity, 98.5%; Taitan, Shanghai, China) (Figure S1) toxicity against adult female* Tetranychus cinnabarinus *[36]. We adopted the bioassay procedure described by Ding et al. [35]. A total of 30 adult female mites (3–5 days old) were briefly placed on their backs on double-sided tape on glass. The mites were then dipped into each test solution for 5 s. Each dose was performed in triplicate. The use of sterile distilled water with 0.1% (v/v) Tween 80 and 3% (v/v) acetone was designated as control treatment. The mites were observed under an anatomical microscope after 48 h of rearing under controlled growth conditions as described in mite rearing. Mites that exhibited immobility or irregular trembling of legs were considered dead. Lethal and sublethal concentrations for subsequent experiments were determined on the basis of log-probit analysis of concentration–mortality data. RNA-seq was employed to analyze transcriptome changes in* Tetranychus cinnabarinus *treated with median lethal concentration (LC_50_) of scopoletin and the solvent against* Tetranychus cinnabarinus* for 24 and 48 h, respectively. For scopoletin treatment, more than 300 female adults were transferred onto three freshly potted cowpea leaves, which were placed in a small Petri dish containing water. The leaves were sprayed with scopoletin solution with the abovementioned concentration. Sterile distilled water with 0.1% Tween 80 and 3% acetone was used as solvent for the control group. Three Petri dishes from one independent experiment comprised a replicate, and two biological replicates were used for RNA purification and sequencing. All sequencing data were submitted to the GEO website (https://www.ncbi.nlm.nih.gov/geo/) with the accession number GSE92959 (unpublished data).

### 2.3. Total RNA Extraction, cDNA Synthesis, and* TcCHIT* Cloning

Total RNA was extracted from 300 adult (3–5 days old)* Tetranychus cinnabarinus* females. Extraction was performed by using RNeasy® Plus Micro Kit (Tiangen, Beijing, China). To determine RNA quantity, the absorbance at 260 nm and absorbance ratio of OD_260/280_ were measured by using a Nanovue UV-Vis spectrophotometer (GE Healthcare, Fairfield, CT). RNA integrity was further confirmed by 1% agarose gel electrophoresis. Reverse transcription was performed by using a PrimeScript® 1st Strand cDNA Synthesis Kit (Takara, Dalian, China). Synthesized cDNA was stored at −20°C. To obtain full-length* TcCHITs*, specific primers were designed and synthesized (Table S3) on the basis of complete genomic sequences from sister species* Tetranychus urticae* (http://bioinformatics.psb.ugent.be/orcae/overview/Teur). Specific polymerase chain reactions (PCRs) were performed in a C1000™ Thermal Cycler (BIO-RAD, Hercules, CA, USA). PCRs were performed with a 25 *μ*L reaction volume with 2.5 *μ*L 10x PCR buffer (Mg^2+^-free), 2.0 *μ*L dNTPs (2.5 mM), 2.5 *μ*L MgCl_2_ (25 mM), 1 *μ*L cDNA templates, 1 *μ*L each primer (10 mM), 0.2 *μ*L rTaq™ polymerase (TaKaRa), and 14.8 *μ*L ddH_2_O. PCR program was 94°C for 3 min, followed by 35 cycles of 94°C for 30 s, 48°C to 60°C (based on primer annealing temperatures) for 30 s, 72°C extension for 1 min to 2 min (based on the predicted length of amplified products), and a final extension of 10 min at 72°C. Amplified PCR fragments were gel-purified by using a gel extraction mini kit (Tiangen, Beijing, China), ligated into pMD™ 19-T vector (Takara, Dalian, China) and then transformed into Trans5*α* competent cells of* Escherichia coli *(Tiangen, Beijing, China). Recombinant plasmids were sequenced at the Beijing Genomics Institute (Beijing, China).

### 2.4. Gene Characterization and Phylogenetic Analysis

Nucleotide sequences of* TcCHITs *were edited by DNAMAN 5.2.2. The deduced amino acid sequences of 12 CHIT proteins were aligned with ClustalW program [31, 37]. Molecular weight and isoelectric point of the proteins were calculated by using ExPASy Proteomics Server (http://cn.expasy.org/tools/pi_tool.html) [38]. The signal peptide was predicted by using SignalP 4.1 (http://www.cbs.dtu.dk/service/SignalP/) [39], and the transmembrane region was analyzed by using TMHMM Server (v.2.0) (http://www.cbs.dtu.dk/services/TMHMM/) [40].* N*-glycosylation sites were predicted by using NetNGlyc 1.0 Server (http://www.cbs.dtu.dk/services/NetNGlyc/) [41]. The phylogenetic tree was constructed by using MEGA 5.0 via neighbor-joining method with 1000 bootstrap replicates [42].

### 2.5. dsRNA Synthesis, dsRNA Feeding, and Knockdown of* TcCHIT* Expression by RNAi

A set of T7 RNA polymerase promoter primers (Table S3) were designed to amplify 160–600 bp lengths of target genes to generate PCR products for in vitro transcription and dsRNA production (Table S3).* TcCHITs *and green fluorescent protein* (GFP)* (ACY56286) genes were amplified by PCR. The PCR program was as described in Section 2.3. Recombinant plasmids were used as templates. The* GFP* gene was used as negative control. Amplified segments were gel-purified and used in application of TranscriptAid T7 High Yield Transcription Kit (Thermo Scientific, Lithuania, EU). dsRNAs were further purified by using GeneJET RNA Purification Kit (Thermo Scientific, Lithuania, EU). Size of dsRNA products was determined by 1% agarose gel electrophoresis. Concentration of dsRNAs was determined by using a spectrophotometer. dsRNAs were stored at −70°C. Systemic delivery of* TcCHIT* dsRNAs via leaf-disc feeding was used to knock down* TcCHIT* expression. In this study, to investigate whether knock down of expression of target genes affects transcript levels of nontarget genes via leaf-disc feeding method, the chitin metabolic pathways related to two chitin synthetase genes (tetur03g08510 and tetur08g00170, designated CHS) were detected when a mixture of 12* TcCHIT* dsRNAs was applied. The mites were fed with a mixture of 12 different* TcCHIT* dsRNAs for 48 h.  Figure S2 shows the schematic diagram of artificial feeding of dsRNA. Briefly, cowpea leaves were cut to a feeding arena (2 cm in diameter) and dehydrated via incubation at 60°C for 3–5 min. The leaves were then treated with diethylpyrocarbonate- (DEPC-) water, dsRNA-*GFP*, or* TcCHIT* dsRNAs (1000 ng/*μ*l) for 3-4 h at room temperature. After complete absorption of liquids, the leaves were placed on wet filter paper. The leaf discs were then placed on water-saturated sponges. Thirty female adults (3–5 days old and starved for 24 h) were placed on each pretreated leaf-disc. The leaf discs were then placed upside down on Petri dishes (7 cm in diameter) to prevent mites from escaping. The dsRNA-treated leaf discs, which were infested with* Tetranychus cinnabarinus*, were placed under controlled growth conditions as described in Section 2.1. The mites were finally collected for subsequent experiments 48 h after feeding.

### 2.6. Quantitative Real-Time PCR (qPCR)

To detect* TcCHIT *expression throughout the different life stages of mites, approximately 2000 eggs, 1500 larvae, 800 nymphs, and 200 adults were collected per sample in triplicate. To quantify* TcCHIT *expression in response to scopoletin and DFB exposure, we collected 200 female adults per sample in triplicate. To examine the effects of scopoletin and DFB exposure on* TcCHIT *expression, female adults were treated with scopoletin or DFB, with 0.1% (v/v) Tween 80 and 3% (v/v) acetone as surfactant. As in the slip-dip assay, LC_10_, LC_30_, and LC_50_ of scopoletin and LC_50_ of DFB corresponded to 0.099, 0.374, 0.938, and 0.477 mg/mL, respectively. For scopoletin and DFB exposure experiment, we adopted a slightly modified version of the leaf-disc dipping method described by Michel et al. [43]. More than 200 female adults (3–5 days old) were briefly transferred to three freshly potted cowpea leaves in a small Petri dish with water. Each detached cowpea leaf was dipped for 5 s in the test solution with the abovementioned concentration. When the liquid had dried around the mites, they were subjected again under the abovementioned conditions. Sterile distilled water with 0.1% Tween 80 and 3% acetone was then used as the control treatment (CK). After a 24 h interval, only surviving female adult mites from the treated and control groups were collected and frozen at −80°C for RNA extraction. Each experiment was performed at least in triplicate and utilized independent biological samples. To examine the effectiveness of RNAi, approximately 200 female adult mites were collected per sample 48 h after dsRNA feeding. Samples were prepared in triplicate. The specific primers used for qPCR of* TcCHITs *were designed by using Primer 3.0 (http://frodo.wi.mit.edu/) (Table S3) [44].* RPS18 *(FJ608659) was used as the stable reference gene for all qPCR assays (Table S3) [45]. qPCR was performed by using a Mx3000P thermal cycler (Agilent Technologies, Inc., Wilmington, NC, USA) on 20 *μ*L reaction mixtures containing 1 *μ*L cDNA template (200 ng/*μ*L), 10 *μ*L iQ™ SYBR® Green Supermix (BIO-RAD, Hercules, CA, USA), 1 *μ*L of each gene-specific primer (0.2 mM), and 7 *μ*L ddH_2_O. The optimized qPCR protocol used for amplification was 95°C for 2 min, followed by 40 cycles of denaturation at 95°C for 15 s, 60°C for 30 s, and elongation at 72°C for 30 s. Melt curve analyses (from 60°C to 95°C) were performed to ensure consistency of amplified products. Quantification of expression level was analyzed using 2^−ΔΔCt^ method [46].

### 2.7. Susceptibility Test of* Tetranychus cinnabarinus* to Acaricides after RNAi of* TcCHITs*

Sublethal doses of scopoletin and DFB (LC_30_ and LC_50_ of scopoletin and DFB, resp.) were applied in bioassays. We also adopted the slip-dip method described above and the detailed bioassay procedure described by Ding et al. [35]. LC_30_ and LC_50_ values of two acaricides were used as diagnostic doses to compare changes in susceptibility to acaricides in* Tetranychus cinnabarinus *48 h after feeding of* TcCHIT *dsRNAs.

### 2.8. Statistical Analysis

All experiments included at least three biological replicates. Differences in expression levels of* TcCHITs* during the four developmental stages and mortality rates were analyzed by one-way analysis of variance, followed by Duncan's multiple tests in SPSS (v.16.0, SPSS Inc., Chicago, IL, USA), at alpha = 0.05.

## 3. Results

### 3.1. Analysis of Acaricidal Toxicity

Table 1 presents the LC_50_ values calculated for the two acaricides against adult* Tetranychus cinnabarinus*. Estimated LC_50_ values of scopoletin and DFB reached 0.938 and 0.477 mg/mL, respectively. These results showed that DFB exhibited more significant acaricidal efficiency compared with scopoletin. However, LC_50_ of scopoletin indicated its excellent toxic effects as a botanical acaricide.

### 3.2. cDNA Cloning and Characterization of* TcCHITs*

**Figure 1:**
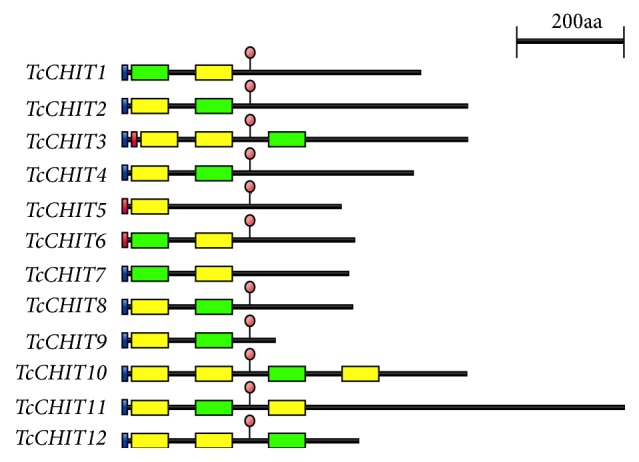
*Domain architecture of putative chitinases of T. cinnabarinus.* Blue boxes: signal peptide; yellow boxes: catalytic domain; green boxes: chitin-binding domain; red boxes: transmembrane span; red circles: the* N*-glycosylation sites; lines: linker regions.

The deduced amino acid sequences and full-length cDNAs of 12* TcCHITs*, which contained open reading frames (ORFs), were deposited in GenBank under the accession numbers indicated in Table 2. Table 2 summarizes the lengths of deduced amino acid sequences, predicted protein molecular weights, and theoretical isoelectric points. A signal peptide was detected at the N-terminal end of* TcCHIT1*,* TcCHIT2*,* TcCHIT3*,* TcCHIT4*,* TcCHIT7*,* TcCHIT8*,* TcCHIT9*,* TcCHIT10*,* TcCHIT11*, and* TcCHIT12 *(Figure 1). Meanwhile,* TcCHIT1*,* TcCHIT2*,* TcCHIT3*,* TcCHIT6, TcCHIT7*,* TcCHIT8*,* TcCHIT9*,* TcCHIT10*,* TcCHIT11*, and* TcCHIT12 *were predicted to contain a chitin-binding domain (Figure 1).* TcCHIT1*,* TcCHIT2*,* TcCHIT4*,* TcCHIT5*,* TcCHIT6*,* TcCHIT7*,* TcCHIT8*, and* TcCHIT9 *were predicted to include one catalytic domain;* TcCHIIT10 *comprised three catalytic domains; and* TcCHIT3, TcCHIT11*, and* TcCHIT12 *comprised two catalytic domains. Furthermore,* TcCHT3*,* TcCHIT5*, and* TcCHIT6 *were predicted to contain one transmembrane span domain. These genes, except for* TcCHIT7*, were also observed to possess potential* N*-glycosylation sites (Figure 1).

### 3.3. Phylogenetic Analysis of* TcCHITs*

**Figure 2:**
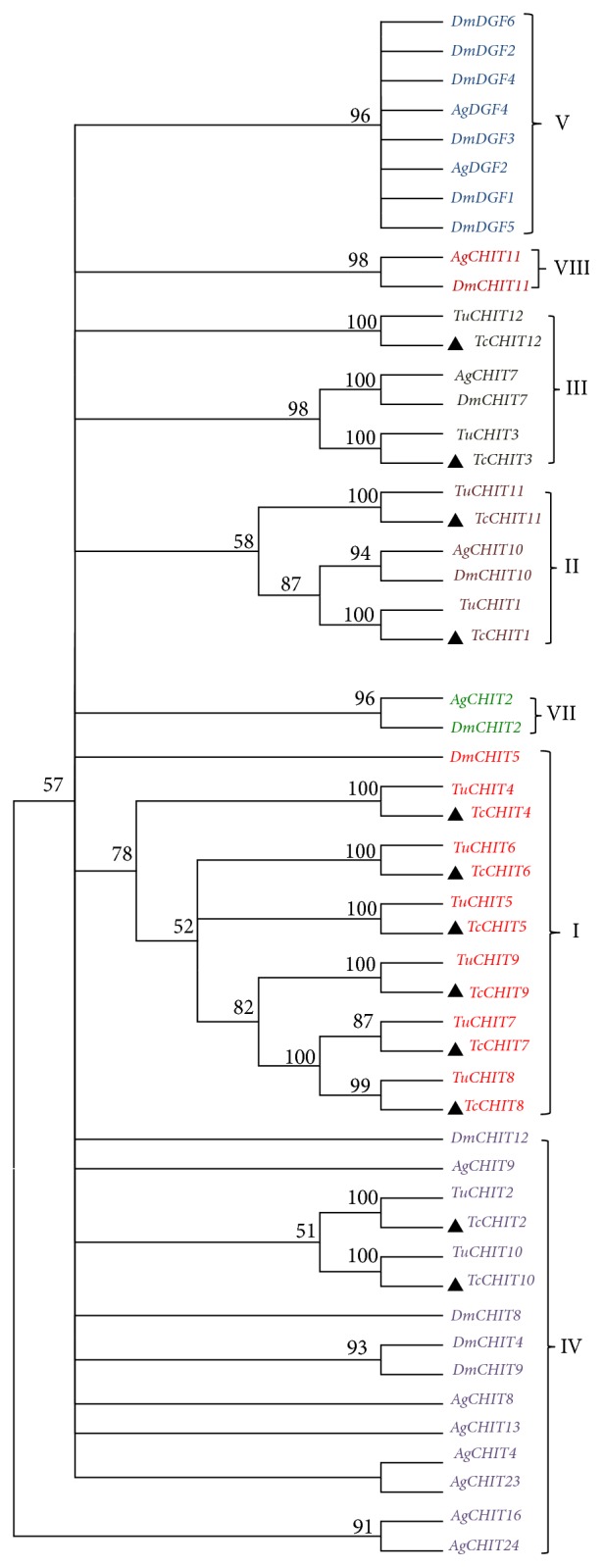
*Phylogenic analysis of TcCHITs.* Maximum likelihood tree constructed by MEGA 5.0. Phylogeny testing was conducted via the bootstrap method with 1000 replications. Sequences used for constructing the tree are listed in Supplementary Table S4.

Phylogenetic analysis was performed by using MEGA 5.0 with the maximum likelihood method on the basis of deduced amino acid sequences of* TcCHITs *and other known CHIT proteins, including orthologs from the family of* Tetranychus urticae*,* Anopheles gambiae*, and* Drosophila melanogaster*. All CHIT sequences, which possess complete ORFs, were obtained from the* Tetranychus urticae *genome and the National Center for Biotechnology Information (Bethesda, MD) (https://www.ncbi.nlm.nih.gov/) (Table S4). Results of phylogenetic analysis revealed that CHIT genes for* Tetranychus cinnabarinus* can be divided into four groups (Figure 2):* TcCHIT4*,* TcCHIT5, TcCHIT6, TcCHIT7, TcCHIT8*, and* TcCHIT9 *under Group I CHITs;* TcCHT1*,* TcCHIT11 *in Group II CHITs;* TcCHIT3, TcCHIT12 *under Group III CHITs;* TcCHIT2, TcCHIT10* under Group IV CHITs. CHIT genes from* Tetranychus cinnabarinus *and* Tetranychus urticae *clustered into the CHIT family and shared a single clade (Figure 2). This result suggests that* TcCHITs *and* TuCHITs* are evolutionarily related and possibly share similar physiological functions.

### 3.4. Expression Patterns of* TcCHITs* in Different Developmental Stages and upon Acaricide Treatment

**Figure 3:**
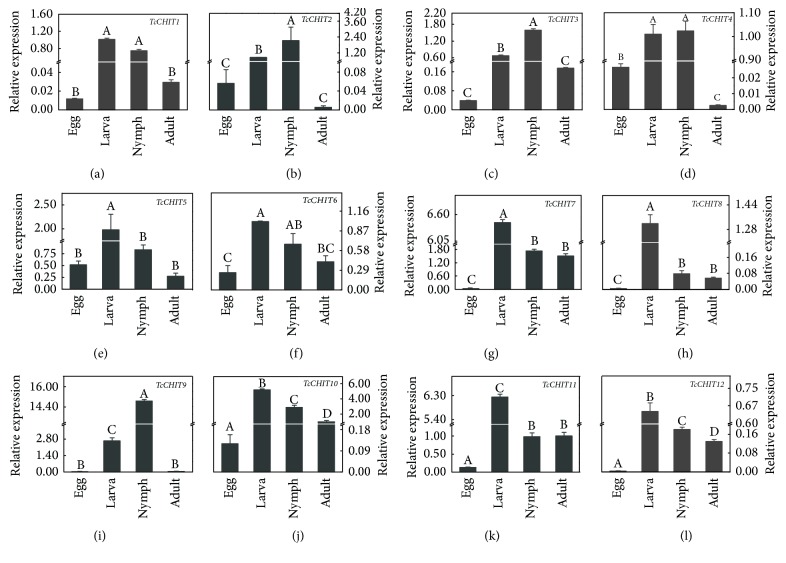
*RT-qPCR evaluation of the developmentally specific expression patterns of the 12 CHIT genes in T. cinnabarinus.* The following life stages were analyzed: egg, larvae, nymph, and adult. Error bars represent the standard error of the calculated mean based on three biological replicates. Different letters on the error bars indicate significant difference according to Duncan's multiple tests (alpha = 0.05), that is, no statistical difference between “A” and “A”; significant difference among “A,” “B,” “C,” and “D.”* RPS18* was used as the reference gene. (a) to (l) were* TcCHIT1~12*, respectively.

qPCR was performed to evaluate* TcCHIT *gene expression levels during different developmental stages (egg, larva, nymph, and adult) and upon acaricidal treatment. Results showed that the 12 CHIT genes* (TcCHIT1 to -12)* were expressed throughout all life stages, suggesting the involvement of* TcCHITs *in biological processes in all developmental and growth stages. Specifically,* TcCHITs *were highly expressed during the larval and nymphal stages compared to in other developmental stages;* TcCHIT *expression levels were the lowest during the egg stage (Figure 3). Statistical analysis suggests that relative expression levels of* TcCHITs *totaled 0.012, 0.056, 0.038, 0.027, 0.514, 0.254, 0.029, 0.004, 0.019, 0.120, 0.126, and 0.004 during the egg stage; 1.009, 0.845, 0.677, 1.009, 1.982, 1.006, 6.434, 1.318, 2.648, 5.192, 6.243, and 0.650 during the larval stage; 0.745, 2.130, 1.583, 1.024, 0.834, 0.672, 1.730, 0.078, 14.873, 2.902, 0.981, and 0.181 during the nymphal stage; and 0.029, 0.006, 0.175, 0.002, 0.275, 0.416, 1.504, 0.057, 0.051, 1.000, 1.000, and 0.129 during the adult stage (Figure 3).

**Figure 4:**
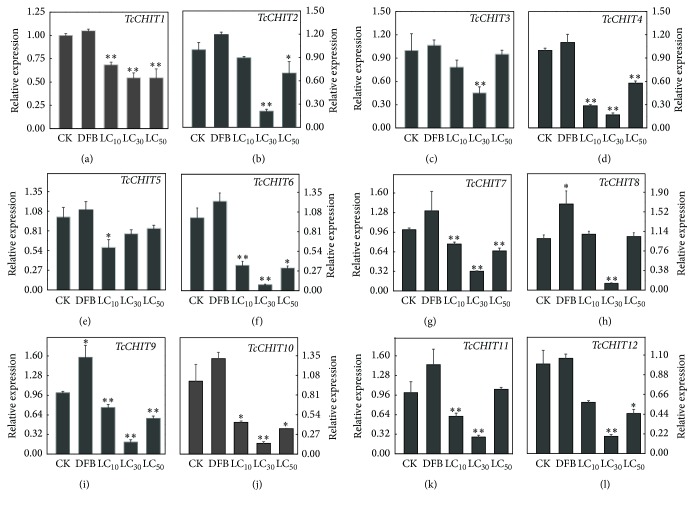
*Expression profiles of TcCHITs transcripts after scopoletin treatment for 24 h at three different concentrations.* Error bars represent the standard error of the calculated mean based on three biological replicates. Water containing 0.1% Tween-80 was used as the control treatment (CK). LC_50_ of DFB was used as the positive control. Asterisk (*∗*) on the error bar indicates a significant difference between the treatment and group (CK) according to* t*-tests, (^*∗*^*p* < 0.05) or (^*∗∗*^*p* < 0.01).* RPS18* was used as the reference gene. (a) to (l) were* TcCHIT1~12*, respectively.

Results of scopoletin treatment experiment showed that, compared with the genes in the control group, all 12 CHIT genes* (TcCHIT1 to -12)* were downregulated after 24 h of exposure to scopoletin (Figure 4). Statistical analysis suggests that compared with expression levels of the control (CK), relative expression levels of* TcCHITs *were 1.5-, 1.1-, 1.3-, 3.5-, 1.7-, 2.9-, 1.3-, 0.9-, 1.3-, 2.3-, 1.6-, and 1.7-fold lower at LC_10_ doses of scopoletin; 1.8-, 4.8-, 2.3-, 6.0-, 1.3-, 12.4-, 2.3-, 7.7-, 5.2-, 6.7-, 3.7-, and 5.2-fold lower at LC_30_ doses of scopoletin; and 1.8-, 1.4-, 1.1-, 1.7-, 1.2-, 3.2-, 1.1-, 1.0-, 1.7-, 2.9-, 0.9-, and 2.2-fold lower at LC_50_ doses of scopoletin. However, relative expression levels of all the 12 CHIT genes were upregulated and were 1.1-, 1.2-, 1.1-, 1.3-, 1.1-, 1.2-, 1.3-, 1.7-, 1.6- 1.3-, 1.5, and 1.1-fold higher than those in the control (CK) after treatment with DFB at LC_50_.

### 3.5. RNAi via dsRNA Knockdown

**Figure 5:**
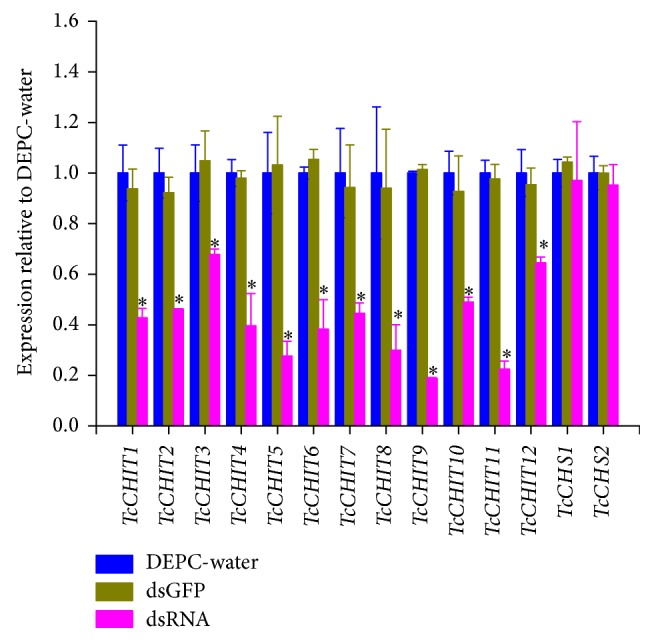
*Quantitative PCR detection of target gene expression after dsRNA-TcCHITs feeding relative to expression levels after DEPC-water treatment.* dsGFP was adopted as negative control.* RPS18* was used as the reference gene. Asterisk (*∗*) on the error bar indicates a significant difference between the treatment and group (DEPC-water) according to* t*-tests, *p* < 0.05.

**Figure 6:**
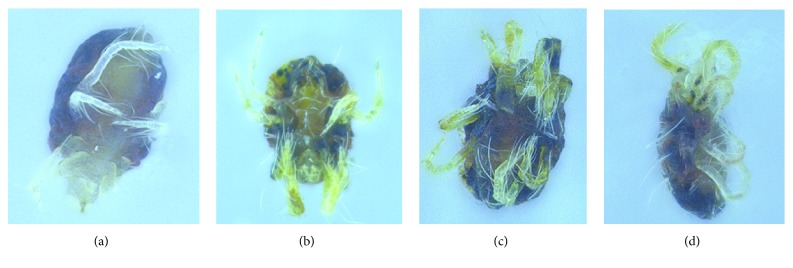
*The phenotypes of T. cinnabarinus after DEPC-water exposure (a), dsRNA-TcCHITs feeding (b), scopoletin (c), and DFB (d) exposure for 48 h at the adult stage.* Adult mites were treated with the LC_50_ of scopoletin and DFB. Note that dsRNA-*TcCHITs *feeding (b), scopoletin (c), and DFB (d) exposure exhibited similar death symptoms: the adult individuals integument shrinkage or damage.

**Figure 7:**
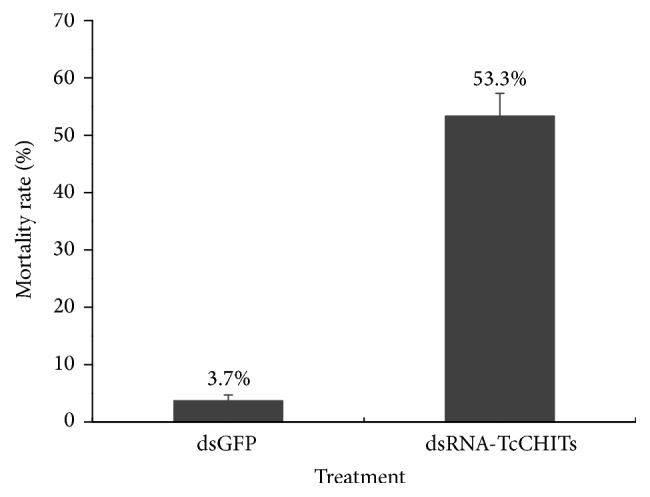
*Mortality rate of T. cinnabarinus after being artificially fed with dsGFP or dsRNA-TcCHITs at the adult stage after 48 h.* Means were compared by* t*-tests, *p* < 0.05 (*n* = 3, including 180 mites).

To validate existence of offsite effects, expressions of all 12 CHIT genes and 2 CHS genes were detected when a mixture of 12* TcCHIT* dsRNAs was applied (Figure 5). mRNA expressions of* TcCHITs* significantly decreased but not those of* TcCHSs* when* TcCHIT* dsRNAs were applied at the adult stage in mites (Figure 5). Results showed that transcript levels of* TcCHITs* significantly decreased to 57.30%, 53.87%, 32.23%, 60.37%, 72.32%, 61.78%, 55.49%, 70.14%, 81.16%, 51.02%, 77.57%, and 35.50% compared with transcript levels of* TcCHITs* after DEPC-water treatment (Figure 5). No significant difference in transcript efficiency existed between the two controls (water and dsGFP) (Figure 5). These results indicate the absence of offsite effect of RNAi experiments in this study. At 48 h after feeding of* TcCHIT *dsRNAs, 53.3% of mites died because of integument shrinkage or damage (Figures 6(b) and 7). By contrast, the mites fed with dsGFP showed 4.7% mortality. These results reveal successful knockdown of* TcCHIT *transcripts by RNAi in* Tetranychus cinnabarinus*.

**Figure 8:**
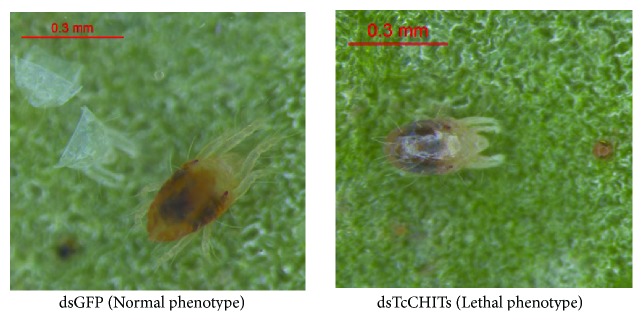
*The phenotypes of T. cinnabarinus after being artificially fed with dsGFP or dsRNA-TcCHITs at the larval stage after 72 h.* The artificial feeding of dsTcCHITs resulted in a lethal phenotype: the larva individuals failed molting or undergoing dysecdysis, whereas mites fed with dsGFP developed normally.

**Figure 9:**
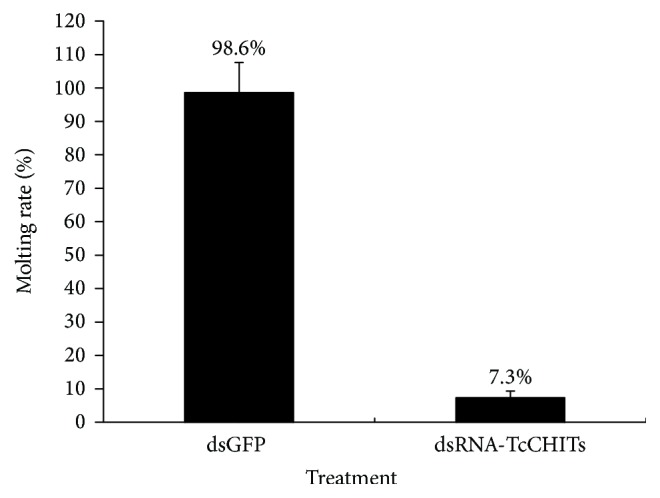
*Molting rate of T. cinnabarinus after being artificially fed with dsGFP or dsRNA-TcCHITs at the larval stage after 72 h.* Means were compared by* t*-tests, *p* < 0.05.

To explore the biological functions of* TcCHITs*, RNAi method was applied to knock down* TcCHITs *expression at the larval stage. Consequently, at 48 h after dsRNA feeding, 92.7% of mites died because of failure to molt or to undergo dysecdysis (Figures 8 and 9, resp.). By contrast, the mites fed with dsGFP exhibited only 1.4% mortality. At 72 h after dsRNA feeding, the remaining mites still failed to molt after treatment with* TcCHIT *dsRNAs, whereas all the mites in the control group successfully turned into nymphs. At 72 h after dsRNA feeding, molting rate reached 7.3% during treatment of* TcCHITs* dsRNAs and totaled 98.6% in dsGFP treatment (Figure 9).

### 3.6. Susceptibility Test of* Tetranychus cinnabarinus* to Acaricides after RNAi of* TcCHITs*

**Figure 10:**
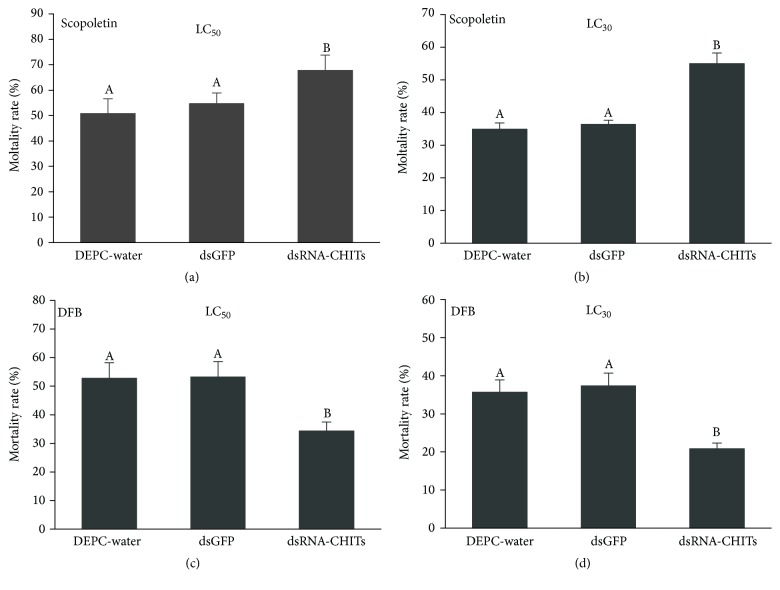
*Knockdown of TcCHITs expressions increased susceptibility to scopoletin and reduced susceptibility to DFB in mites*. (a, b) Mortality of* TcCHITs*-silenced* T. cinnabarinus *to scopoletin at LC_50_ and LC_30_, respectively. (c, d) Mortality of* TcCHITs*-silenced* T. cinnabarinus *to DFB at LC_50_ and LC_30_, respectively. dsGFP was adopted as negative control. Error bars represent the standard error of the calculated mean based on three biological replicates. Different letters on the error bars indicate significant difference according to Duncan's multiple tests (alpha = 0.05) that is, no statistical difference between “A” and “A”; significant difference between “A” and “B”.

Susceptibilities to the two acaricides at 48 h after* TcCHITs *dsRNA feeding at the adult stage were detected by the slip-dip method.* TcCHITs *transcripts in the LC_50_ and LC_30_ assays of scopoletin were knocked down by RNAi in* Tetranychus cinnabarinus*. Mortality significantly increased to 16.92% and 20.12% in the mites fed with* TcCHITs *dsRNAs compared with mites treated with DEPC-water (Figure 10). Contradictory results were generated when* TcCHITs *transcripts in the LC_50_ and LC_30_ assays of DFB were knocked down by RNAi in* Tetranychus cinnabarinus*. Mortality significantly decreased to 18.43% and 14.88% in the mites fed with* TcCHITs *dsRNAs compared with those treated with DEPC-water (Figure 10). No significant difference in mortality existed between DEPC-water and dsRNA-*GFP *treatments (Figure 10). These results demonstrate that RNAi of CHIT genes increased susceptibility of* Tetranychus cinnabarinus *to scopoletin but reduced that to DFB. These results indicate that CHIT genes may play a crucial role in the acaricidal effects of scopoletin and DFB.

## 4. Discussion

As an important phenolic phytoalexin in plants, scopoletin features numerous pharmacological activities, such as antitumor activity. Scopoletin can affect and disrupt growth, proliferation, metastasis, and metabolism of tumor cells and induce apoptosis [47, 48]. However, the potential acaricidal mechanism of scopoletin as a plant-derived acaricide against* Tetranychus cinnabarinus* remains unknown.

Identification and characterization of CHIT genes from mites will aid in determining the involvement of CHITs in responses of mites to specific acaricides. Findings of the present study will also help us to better understand the biological functions of CHITs. We cloned and characterized 12 full-length CHIT cDNAs in* Tetranychus cinnabarinus *(*TcCHIT1 *to* -6*; Wang et al. [26]). In 1993, full-length cDNA of the first insect CHIT gene was cloned and identified from the tobacco hornworm* Manduca sexta* [49]. Since then, CHIT genes of various insects have been cloned and identified. The encoded CHITs in* Tribolium castaneum* have been divided into eight subgroups on the basis of sequence similarity and domain architecture [50]. Structural analysis of* TcCHITs *demonstrated that these genes possess a multidomain structural organization, which includes 1–3 catalytic domains (GH-18 domain), 0-1 cysteine-rich chitin-binding domains (peritrophin-A domain/CBM-14 domain), and serine/threonine-rich regions that can be heavily glycosylated (Figure 1).* TcCHITs* are predicted to feature a signal peptide (*TcCHT1, TcCHT2, TcCHT3, TcCHT4, TcCHT7, TcCHT8, TcCHT9, TcCHT10, TcCHT11, *and* TcCHT12*) or a transmembrane (*TcCHT3, TcCHT5, *and* TcCHT6*) span domain as they are targeted either to the extracellular space or sorted into the plasma membrane, in both cases facing carbohydrates of the extracellular matrix (Figure 1). Differences in their domain architecture indicate distinctive biological functions for specific CHITs. For example, Xia et al. [34] reported that* CHIT5*,* TcCHT7*, and* TcCHT10*, which are also expressed at all developmental stages, play critical roles in digesting the old pupal cuticle, wing/elytra extension, and molting in* Tribolium castaneum*, respectively.

*TcCHIT* transcripts were detected in all four tested developmental stages of* Tetranychus cinnabarinus*, indicating that the CHIT gene family plays an important role during the entire life cycle of mites. However,* TcCHIT* expression levels during larval and nymphal stages were significantly higher than in other developmental stages of* Tetranychus cinnabarinus*, agreeing with the results of Yang et al. [51]. Our present results showed that the CHIT gene family exhibits different developmental patterns of expression in* Tetranychus cinnabarinus*. Expression level of* TcCHIT9* during the larval and nymphal stages was approximately 250-fold higher than those during the egg and adult stages, whereas* TcCHT3*,* TcCHT7*,* TcCHT10*, and* TcCHT11* were approximately 10-fold higher. Differences in expression levels may be related to the structure and function of CHIT genes. Different developmental patterns of expression indicate functional specialization in the CHIT gene family of* Tetranychus cinnabarinus* during molting. In addition, RNAi studies in* Tribolium castaneum* have provided strong experimental evidence for different developmental patterns of expression and tissue-specific expression of different CHIT genes [34].

Results of the present study further showed that 24 h after scopoletin exposure,* TcCHIT *expression levels were downregulated and that the challenge of inhibiting* TcCHIT1*,* TcCHT6*,* TcCHT7*,* TcCHT9, *and* TcCHT10 *expressions was more significant than that of inhibiting the remaining CHIT genes in* Tetranychus cinnabarinus*. Specifically, statistical analysis suggests that* TcCHIT *expression levels were downregulated more significantly with LC_30_ dose of scopoletin than with LC_10_ and LC_50_ doses of scopoletin than those of the control. This result suggests the possible benefits of using appropriate doses of scopoletin. This study also showed that* TcCHITs *expression levels were significantly downregulated during the adult stage of mites. The preceding results indicate that the acaricidal mechanism of scopoletin may degrade chitin biodegradation in* Tetranychus cinnabarinus* by decreasing CHIT gene expression. However, relative expression levels of* TcCHIT8* and* TcCHT9 *were significantly upregulated after DFB treatment (positive control). Several studies have shown that the action mechanism of DFB may occur through direct inhibition of chitin synthase activity; inhibition of zymogen activation process; activation of CHITs; interference with hormonal balance; interference with nerve-secreting cells of the brain; and interference with nucleic acid, protein synthesis, and metabolism in several different insect species [52]. However, the acaricidal mechanism of DFB remains unknown. These results suggest that* TcCHITs *play an essential role in acaricidal mechanisms of scopoletin and DFB in* Tetranychus cinnabarinus*.

In this study, we employed RNAi to investigate the biological roles of mite CHIT genes in* Tetranychus cinnabarinus*. RNAi has become an increasingly common method to knock down expression of genes of interest in insects and mites. RNAi also features potential applications in screening and identification of pharmaceutical targets. Previous studies have demonstrated that expressions of specific CHIT genes in* Tribolium castaneum *can be knocked down by microinjection assays with gene-specific RNAi [34]. For example, Xia et al. [34] reported that specific knockdown of* CHT10* transcripts of* Tribolium castaneum, *which contains multiple catalytic domains, prevented embryo hatching, larval molting, pupation, and adult metamorphosis. In this study, results of qPCR analyses showed that* TcCHIT *transcript levels significantly decreased to 30%–80% in mites 48 h after feeding with* TcCHITs *dsRNAs. In this study,* TcCHITs* silencing in larvae and adult samples resulted in high mortality rate. These results not only demonstrated successful dsRNA-mediated knockdown of* TcCHITs *transcripts but also showed suitability of dsRNA delivery via the leaf-disc method for RNAi in* Tetranychus cinnabarinus*.

Several studies have shown that activation or inhibition of CHITs kills insects or mites. Ding et al. [53] reported that mortality of* M. sexta* larvae was higher when treated with a sublethal dose of* Bacillus thuringiensis *(Bt) toxin on transgenic tobacco lines that express* M. sexta *CHIT (Group I). This result suggests that overexpression of CHIT increases susceptibility of* M. Sext*a to Bt. In addition, Sakuda et al. [25] showed that allosamidin strongly inhibits insect CHITs and exerts insecticidal effects by preventing molting of insect larvae and pupae. These results indicate that inhibition of CHITs can lead to insect death. Our results showed that* TcCHITs* play a similar role in acaricidal mechanisms of scopoletin and DFB. Expressions of* TcCHITs *were downregulated 24 h after scopoletin exposure. Specifically, scopoletin susceptibility increased when* TcCHITs *in LC_50_ and LC_30_ assays were suppressed via RNAi. These results suggest that the acaricidal mechanism of scopoletin may transpire by inhibition of expressions of CHIT genes. However,* TcCHIT *expressions were upregulated 24 h after DFB treatment. Meanwhile, DFB susceptibility decreased when* TcCHIT *transcripts were knocked down by RNAi in* Tetranychus cinnabarinus*. These results indicate that the acaricidal mechanism of DFB may transpire by activation of CHIT gene expression. The function of CHIT genes of* Tetranychus cinnabarinus* has not been reported at present. However, Zhang et al. [27] reported that CHITs are closely related to insect molting in* Tribolium castaneum*, suggesting that acaricidal mechanism of scopoletin and DFB possibly prevents mites from undergoing normal growth and development by destroying the integument of* Tetranychus cinnabarinus.* This paper is the first to report that knockdown of CHIT gene expression in* Tetranychus cinnabarinus* increases susceptibility to scopoletin but reduces that to DFB.

## Figures and Tables

**Table 1:** Toxicity of acaricides against adult *T. cinnabarinus *after 48 h of exposure time.

Acaricide	*N*	LC_50_ (mg·mL^−1^)^a^ 95% CI^b^	Slope (±SE)	*χ* ^2^ ^c^	*P*
Scopoletin	540	0.938 (0.576~2.292)	1.314 (±0.15)	6.321	0.097
DFB	540	0.477 (0.118~0.902)	2.254 (±0.24)	5.939	0.051

^a^LC_50_: median lethal concentration. ^b^CI: 95% confidence interval. ^c^Chi-square testing linearity, alpha = 0.05.

**Table 2:** Complete sequence information of the 12 CHIT genes of *T. cinnabarinus*.

Gene	Accession numbers	Coding sequence (bp)	Deduced full-length of amino acid	Calculated full-length of molecular (kDa)	Isoelectric point
*TcCHIT1*	KT956964	1632	543	60.8	5.44
*TcCHIT2*	KT956965	1887	628	69.03	8.65
*TcCHIT3*	KT956966	2793	930	104.32	6.23
*TcCHT4*	KT956967	1593	530	59.75	6.07
*TcCHT5*	KT956968	1194	397	45.63	6.12
*TcCHT6*	KT956969	1272	423	49.05	8.27
*TcCHIT7*	KY084261	1233	410	47.34	9.19
*TcCHIT8*	KY084262	1260	419	48.51	9.64
*TcCHIT9*	KY084263	834	277	32.02	8.92
*TcCHT10*	KY084264	1881	627	69.55	6.65
*TcCHIT11*	KY084265	2748	915	99.42	6.46
*TcCHT12*	KY084266	1293	430	48.75	6.98

## Supplementary Materials

Supplementary Materials Table S1. The differentially expressed genes between scopoletin- and solvent-treated mites at 24 h after treatment in RNA-seq. Table S2. The differentially expressed genes between scopoletin- and solvent-treated mites at 48 h after treatment in RNA-seq. Table S3. Primers used for cloning, qPCR, and RNAi. Table S4. Sequences and relevant information for phylogenetic analysis of TcCHITs. Figure S1. Chemical structure of scopoletin (A) and diflubenzuron (DFB) (B). Figure S2. A picture of leaf-disc mediated dsRNA feeding.
