# Supplementary material for: Silencing Chitinase Genes Increases Susceptibility of Tetranychus cinnabarinus (Boisduval) to Scopoletin
Source: Biomed Res Int. 2017 Dec 31;2017:9579736. doi: 10.1155/2017/9579736 (PMC5804380; doi:10.1155/2017/9579736)
Supplement: Supplementary Materials — Table S1. The differentially expressed genes between scopoletin- and solvent-treated mites at 24 h after treatment in RNA-seq. Table S2. The differentially expressed genes between scopoletin- and solvent-treated mites at 48 h after treatment in RNA-seq. Table S3. Primers used for cloning, qPCR, and RNAi. Table S4. Sequences and relevant information for phylogenetic analysis of TcCHITs. Figure S1. Chemical structure of scopoletin (A) and diflubenzuron (DFB) (B). Figure S2. A picture of leaf-disc mediated dsRNA feeding. [file 9579736.f1.zip › Table S3, Figure S1, Figure S2.docx]

**Supplemental materials**

**Silencing chitinase genes increases the susceptibility of *Tetranychus cinnabarinus* (Boisduval) to scopoletin**

Hong Zhou󠅹^£^, Yong-qiang Zhang^£^, Ting Lai, Dan Wang, Jin-lin Liu, Fu-you Guo & Wei Ding*

*Institute of Pesticide Science, College of Plant Protection, Southwest University, Chongqing 400715, P.R. China*

**Table S3** Primers used for cloning, qPCR, and RNAi

| Experiments | GenBank No. | Genes | Forward Primer (5′-3′) | Reverse Primer (5′-3′) | Product length (bp) |
| --- | --- | --- | --- | --- | --- |
| cloning | KT956964 | TcCHIT1 | ATGTTGCCATCTTTAATCATCT | TCAACAATCAATTCCTTCGAAA | 1632 |
|  | KT956965 | TcCHIT2 | ATGACCACAATTGTTAATTATT | TTATTTGCGATTTTTTCCATCC | 1887 |
|  | KT956966 | TcCHIT3 | ATGAGTGGGACATCGGTGATCA | TTATTTGGGTGCTGCTGCAGTT | 2793 |
|  | KT956967 | TcCHIT4 | ATGGCTAGAATGATAAACCTGA | TTAAATGTTGTTACAATTGGCA | 1593 |
|  | KT956968 | TcCHIT5 | ATGAGGAAAAAATTGACTTTTG | TCAACGTTTGAGATCATCGTTA | 1194 |
|  | KT956969 | TcCHIT6 | ATGAAAATAATGAAGAAACTGG | TCAAGTGAAAAACTTGAGCGTA | 1272 |
|  | KY084261 | TcCHIT7 | ATGAATAGCAGACTTTATTGGA | TCAATATCTAAAGCCAGAGTTG | 1233 |
|  | KY084262 | TcCHIT8 | ATGAATAGCAGACTTTATTGGA | CTAACCTATTGACCAAATCATG | 1260 |
|  | KY084263 | TcCHIT9 | ATGAATCGTAATAGTCCAATCT | TCATGATCGGGGGTTCCACTTC | 834 |
|  | KY084264 | TcCHIT10 | ATGCAGCTATTCAATTTTCATC | TTAAGCTCGCTTTAAAGATGAT | 1881 |
|  | KY084265 | TcCHIT11 | ATGACCCAAGATACTGAAGAAG | TTATAAATAGCAATTGATATTG | 2748 |
|  | KY084266 | TcCHIT12 | ATGGTTAAAGGTTTACTTTTAC | TTAGATTAGTCCTGGAACCATT | 1293 |
| qPCR | FJ608659 | RPS18 | ACGTGCTGGTGAACTTACCGAAGA | TGCCTATTCAAGAACCAAAGTGGG | 99 |
|  | tetur03g08510 | TcCHS1 | GGCTCAGATCAAAGAAGGCC | GAAAGATCAGATCGGTCGCG | 201 |
|  | Tetur08g00170 | TcCHS2 | ACGTTTTCTTGATGCTCGCA | CTTCTCGTGTTCCCCAGCTA | 249 |
|  | KT956964 | TcCHIT1 | GCCCATCATGCACCATTGTA | CCCACCTTGTAAAGCAGCTG | 222 |
|  | KT956965 | TcCHIT2 | CATCATCTCCATCGCCGCCT | GTCAAGACCGGCAAATGTGT | 170 |
|  | KT956966 | TcCHIT3 | TGTCGTCGGAATGGCTACTT | GCTGCACCGTCCTTAAGAAG | 165 |
|  | KT956967 | TcCHIT4 | TCATTGCTGTTGGTGGTTGG | TCGATCAGTAGCTCCAGGAT | 164 |
|  | KT956968 | TcCHIT5 | GGTTCTGCCGATGTTCACAG | AACATGCCGCTTTCATTGGT | 245 |
|  | KT956969 | TcCHIT6 | GCCTACTTGAGCTTCTGTGC | ACGGAGATGGACGATAAGTT | 172 |
|  | KY084261 | TcCHIT7 | TGCGTTGAGGTTAAAGTGGC | TCGACGACACTCCAGGATAC | 197 |
|  | KY084262 | TcCHIT8 | TGAGCCAGGATTCTTGACTT | CCGCTAAGCTCTCGTCATCT | 188 |
|  | KY084263 | TcCHIT9 | TGGCTTGAGAACGGAGCTAA | TAGCTCTCCACCTCTCCATG | 159 |
|  | KY084264 | TcCHIT10 | GCAAAGGAGCAGGATAACGG | AACCCATTGTCGACCAGAGT | 186 |
|  | KY084265 | TcCHIT11 | TTACCACCACCTCCACAACA | CGGCCCATTTGTCAATCGAT | 249 |
|  | KY084266 | TcCHIT12 | ATTGGAGGCTGGTCAGAACA | TGAGAACGACAATCACCATT | 170 |
| RNAi | ACY56286 | GFP | taatacgactcactatagggTCGGCGGCAATCCTGATCAA | taatacgactcactatagggTCACAGGGTAAAATTCAGCA | 476 |
|  | KT956964 | TcCHIT1 | taatacgactcactatagggCGAATTGTCGCCTTGAAACG | taatacgactcactatagggGGGAACATCATAACCAGCAT | 357 |
|  | KT956965 | TcCHIT2 | taatacgactcactatagggGGTCTTGCTGGAATAGGGATT | taatacgactcactatagggCGGAAGGCTTACCGACTGAA | 424 |
|  | KT956966 | TcCHIT3 | taatacgactcactatagggTATTGTTGGCAGTGGGAGGTT | taatacgactcactatagggTTCGTCCATAAGTTGGTAGGC | 522 |
|  | KT956967 | TcCHIT4 | taatacgactcactatagggTAAGAAATGGATTGGAGGAG | taatacgactcactatagggTGATGTGGGTTTAGATGTTG | 502 |
|  | KT956968 | TcCHIT5 | taatacgactcactatagggCAACTTGGGCAGCCTTTCGT | taatacgactcactatagggTGCAGGTACAGCGGCAGATA | 473 |
|  | KT956969 | TcCHIT6 | taatacgactcactatagggCCCAGGGTCAACAGAGCATC | taatacgactcactatagggTAGTTTCGTCATCCCATTCT | 542 |
|  | KY084261 | TcCHIT7 | taatacgactcactatagggAGTGGCTATGATGTCCCTCG | taatacgactcactatagggTTGCCAGCTATTCCAACG | 305 |
|  | KY084262 | TcCHIT8 | taatacgactcactatagggAACACGCAAAAGTCACCGAT | taatacgactcactatagggTGGTCCATTTTCCCGACTCA | 163 |
|  | KY084263 | TcCHIT9 | taatacgactcactatagggCAAGATGCAGCCGAAGTCTC | taatacgactcactatagggTGACGTGCCAGAACAAATCG | 231 |
|  | KY084264 | TcCHIT10 | taatacgactcactatagggTTCTTCTCGGTGATCTCGGG | taatacgactcactatagggTGGGATTGGCTGGAGGAA | 165 |
|  | KY084265 | TcCHIT11 | taatacgactcactatagggTCTGCAACCAATGGCTCTGA | taatacgactcactatagggCTGTCCCGATAAATCTTC | 480 |
|  | KY084266 | TcCHIT12 | taatacgactcactatagggAATCTCCACTATTTAGCCAACC | taatacgactcactatagggATCATCTCGATCAATGTCCC | 419 |

Figure S1

A B

**Figure S1 Chemical structure of scopoletin (A) and diflubenzuron (DFB) (B)**

**
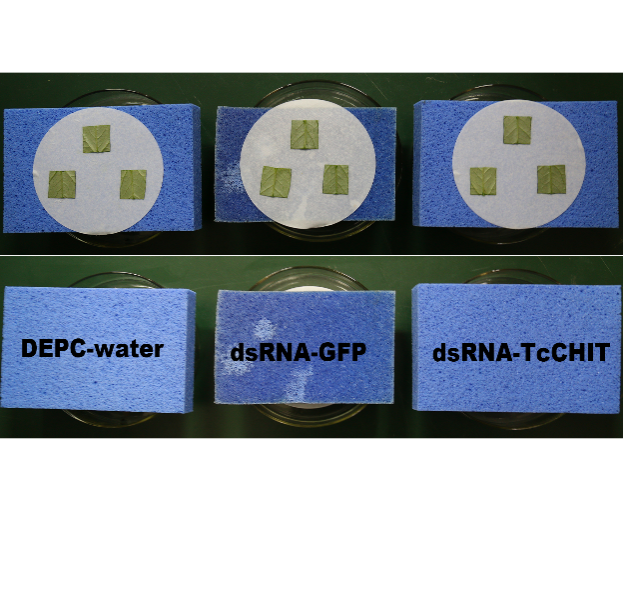
**

**Figure S2. A picture of leaf-disc mediated dsRNA feeding.**
